# Bodyweight Measures and Lifestyle Habits in Individuals with Multiple Sclerosis and Moderate to Severe Disability

**DOI:** 10.3390/jcm10102083

**Published:** 2021-05-12

**Authors:** Moran Livne-Margolin, Itay Tokatly Latzer, Orit Pinhas-Hamiel, Gil Harari, Anat Achiron

**Affiliations:** 1 Sackler School of Medicine, Tel-Aviv University, Ramat Aviv 69978, Israel; moranlivne@gmail.com (M.L.-M.); orit.hamiel@sheba.health.gov.il (O.P.-H.); anat.achiron@sheba.health.gov.il (A.A.); 2 Gastroenterology Department, Sheba Medical Center, Tel Hashomer, Ramat Gan 52621, Israel; 3 Pediatric Neurology Institute, Dana-Dwek Children’s Hospital, Tel Aviv Medical Center, Tel Aviv 60198, Israel; 4 Pediatric Endocrinology and Diabetes Unit, Edmond and Lily Safra Children’s Hospital, Sheba Medical Center, Tel-Hashomer, Ramat Gan 52621, Israel; 5 School of Public Health, Faculty of Social Welfare and Health Sciences, University of Haifa, Haifa 3498838, Israel; gil@medistat.co.il; 6 Multiple Sclerosis Center, Sheba Medical Center, Tel-Hashomer, Ramat Gan 52621, Israel

**Keywords:** expanded disability status scale, overweight, obesity, central obesity, multiple sclerosis

## Abstract

Multiple sclerosis (MS) is a chronic disease marked by progressive disability and decreased mobility over time. We studied whether individuals with MS of higher disability levels will be more overweight/obese as a result of their immobility and/or recurrent steroid treatments. In a prospective study, 130 individuals with MS and significant disability were classified according to the Expanded Disability Status Scale (EDSS) score as belonging to four groups: EDSS 3.0–4.0 (*n* = 31, 24%), EDSS 4.5–5.5 (*n* = 24, 18%), EDSS = 6.0 (*n* = 44, 34%) and EDSS ≥ 6.5 (*n* = 31, 24%). Medical history, body mass index (BMI), waist circumference and the level of engagement in physical activity were obtained. The mean ± standard error age was 55.8 ± 0.5 years, disease duration 18.2 ± 1.0 years and EDSS score 5.5 ± 0.1. Disease duration, the number of steroid courses per disease duration, weight, BMI and physical activity did not differ according to the four disability groups. The mean waist circumference increased significantly with increased severity of EDSS, *p* = 0.03. Increased disability in individuals with MS was not correlated with disease duration, lifestyle habits or overweight/obesity. However, increased disability was associated with central obesity.

## 1. Introduction

Multiple sclerosis (MS) is the most common cause of progressive neurologic disability in young adults [1]. Accumulation of disability over time is associated with motor weakness, imbalance and gait difficulties. Despite the weight loss secondary to progressive loss of muscle mass, limited physical activity may contribute to an overall weight gain and increased prevalence of overweight and obesity [2].

Obesity is a worldwide epidemic in developed and developing countries, and its prevalence is increasing globally [3]. Obesity is a well-known risk factor for a number of comorbidities and is associated with increased risk of dyslipidemia, hypertension, type 2 diabetes mellitus, stroke, cardiovascular disease [4], osteoarthritis and negative effects on the nervous system [5], as well as with increased mortality. Furthermore, obesity is associated with an increased prevalence of autoimmune diseases, such as rheumatoid arthritis, psoriasis and MS [6,7]. This suggests that autoimmune factors may be involved in the etiology of obesity in MS. Indeed, several studies have reported an association between childhood obesity and increased risk of MS in adulthood [8,9]. Specifically, adipokines associated with obesity, such as leptin, adiponectin, resistin and visfatin, were suggested as possible modulators of the immune response [7]. Additionally, this population is frequently treated with high-dose steroids during acute relapses, further increasing the risk for weight gain.

In a longitudinal follow-up study, individuals with MS did not experience age-expected increases in body mass index (BMI) [10]. Similarly, we reported lower rates of obesity and overweight, as assessed by BMI, among individuals with MS and disability than in the general population [11]. However, higher rates of central obesity, as reflected by increased waist circumference, were found among individuals with MS. In the present study, and differently from previous studies, we assessed the occurrence of overweight and obesity among individuals with MS in relation to their various disability categories, taking into account other possible associated variables. We hypothesized that individuals with MS and high disability may be more prone to overweight or obesity, secondary to decreased motor function, immobility and recurrent steroid treatments.

## 2. Methods

### 2.1. Design

This cross-sectional prospective study was conducted in a single tertiary medical center. Individuals with MS who arrived for a neurological follow-up, met the inclusion criteria and consented to participate, underwent anthropometric measurements (weight, height, waist circumference) and a comprehensive neurological examination. Neurologic disability was determined by the Expanded Disability Status Scale (EDSS). The participants were also asked about their physical activity. From the medical charts of the MS Center, computerized data registry, demographic and clinical variables were retrieved, including those relating to sex, age, disease duration and the number of steroid treatments since disease onset. Anthropometric data obtained in this study were partially reported previously [11]. The study was approved by the institutional review board (IRB) committee of Sheba Medical Center (0392-13SMC, 1 July 2013).

### 2.2. Study Population

Study eligibility criteria included individuals diagnosed with MS according to McDonald criteria [12] who were aged 40 to 65 years. We focused on this age group to allow a sufficiently long duration of illness in which obesity could develop. Importantly, those included also had a neurological disability according to an EDSS score ≥ 3.0, confirmed after two consecutive assessments 6 months apart [13]. An EDSS score of 3.0–4.0 represents moderate to significant disability with no impairment in unaided walking for a distance of 500 m without aid or rest. An EDSS score of 4.5–5.5 represents disability that is severe enough to preclude full daily activities; walking ability is in the range of 300 m (EDSS 4.5) to 100 m (EDSS 5.5). An EDSS score of 6.0 signifies severe disability, by which the individual requires a walking aid such as a cane or a crutch to walk a distance of 100 m without rest. An EDSS score of 6.5 represents severe disability, i.e., requiring two walking aids—a pair of canes or crutches—to walk about 20 m without resting.

### 2.3. Anthropometric and Treatment Parameters

BMI was calculated as weight (kg) divided by height squared (m^2^). Normal BMI was defined as 18.5–24.9 kg/m^2^, overweight as a BMI of 25.0–29.9 kg/m^2^ and obesity as a BMI ≥ 30 kg/m^2^ [14]. Waist circumference was measured with a tape measure at the midway between the iliac crest and the lowest rib. Increased waist circumference was defined as above 102 cm for men and above 88 cm for women according to the National Heart, Lung, and Blood Institute of the National Institutes of Health cutoffs [15]. The number of steroid courses was corrected per disease duration.

### 2.4. Lifestyle Parameters

Physical activity was assessed according to the number of sessions/weeks of at least 60-min duration, of any type of physical activity, in the preceding 6 months. Following the 2008 Physical Activity Guidelines of the US Centers for Disease Control, engagement in physical activity was categorized based on duration and frequency as follows: low, <2.5 h per week; target level, ≥2.5 h but <5 h per week; and optimal level, ≥5 h per week [16].

### 2.5. Statistical Analysis

Statistical analysis was performed using SAS^®^ version 9.1 (SAS Institute, Cary, NC, USA). Analyses included descriptive statistics for demographic, clinical and anthropometric data. The Chi-square test was applied to examine variation in frequencies of categorical variables between EDSS groups. Analysis of variance (ANOVA) was used to examine differences in demographic, clinical and anthropometric variables between EDSS groups. All the tests were two-tailed, and a *p*-value of 0.05 or less was considered statistically significant.

## 3. Results

The study cohort comprised 130 individuals with MS of which 72% were females; age (mean ± standard error) 55.8 ± 0.5 years, mean disease duration 18.2 ± 1.0 years and mean EDSS score 5.5 ± 0.1. The mean BMI was 26.5 kg/m^2^; 47% had normal BMI, 35% were overweight and 18% were obese. At the time of assessment, 71 (55%) were treated with immunomodulating agents. The clinical and anthropometric variables of the study participants according to EDSS groups are presented in Table 1. No statistical differences were found between the EDSS groups, in mean age, disease duration, the number of steroid courses corrected to disease duration, weight and BMI. The mean waist circumference increased significantly with increased disability: 94.2 ± 1.6, 96.6 ± 3.2, 96.6 ± 2.2 and 104.4 ± 3.0 for EDSS 3.0–4.0, 4.5–5.5, 6.0 and ≥6.5, respectively, *p* = 0.045. Sixty-six percent of females and 35% of males had central obesity. The occurrence of central obesity increased with increased disability: 11%, 33%, 45% and 45% among males and 59%, 61%, 61% and 85% among females.

Fifty-seven percent of the participants were engaged in some physical activity. Reported engagement in physical activity was similar between the EDSS groups (Table 1).

A weak positive association was demonstrated between EDSS scores and waist circumference (r = 0.2, *p* = 0.02). No correlations were found of EDSS scores with BMI, weight, disease duration, and the number of steroid treatments corrected to disease duration. Likewise, no correlations were found of waist circumference with disease duration or with the number of steroid treatments.

## 4. Discussion

In the current study of individuals with MS and significant disability, increased disability was not associated with overweight or obesity, disease duration, the number of steroid courses, or engagement in physical activity. However, waist circumference was significantly higher among participants with greater disability.

Neurological disability due to MS evolves with disease progression and is associated with significant impairment in activities of daily living due to dysfunctions in motor, sensory, balance and gait functions which decrease mobility. The long-term effects of disability may result in decreased physical activity, a hazardous sedentary lifestyle, consumption of condensed high energy and fast foods, and increased day-time sleep, all resulting in an increased risk for obesity [17]. In addition, the chronicity of MS and the recurrent high-dose steroid treatments over the years, may lead to muscle atrophy, decreased bone mass, increased cardiovascular morbidity, sore ulcers and a predisposition for infections. The well-known adverse effects of steroids further aggravate the secondary morbidities of obesity [18].

In the current study we demonstrated an association of neurological disability with central obesity but not with BMI. This corroborates our previous report that individuals with MS and significant disability (EDSS score ≥ 3.0) were 1.7-fold more likely to be overweight and with obesity than age- and gender-matched Israelis [11]. It is of note that central obesity was already present in two-third of females with EDSS 3.0–4.0, and in 85% of females with EDSS ≥ 6.5. Among males, central obesity increased from 11% of those with EDSS 3.0–4.0 to 45% of those with EDSS ≥ 6.5.

An association of mild to moderate neurological disability with higher waist circumference was reported among 110 individuals with MS [19]. Similarly, a large study of individuals with MS demonstrated a strong association of increased central obesity with severe disability [20]. However, in that study, waist circumference and disability were self-reported and not determined objectively. Our findings corroborate these findings according to the severity of disability, after measuring weight, height and abdominal circumference.

Central obesity is the cornerstone of the metabolic syndrome. Indeed, we previously reported the metabolic syndrome among 30% of individuals with MS [11,21]; this compares with a 10.6% prevalence among adults in the general population [11,21]. Central obesity has been demonstrated as a major risk factor for coronary heart dis-ease, and to be strongly associated with other comorbidities such as increasing insulin resistance, hypertension and hypercholesterolemia [22]. Disability in individuals with MS was also found to be associated with insulin resistance [19].

Importantly, significantly lower BMI relative to the general population was re-ported among individuals with various neurological disabilities; however, changes in body composition resulted in central adiposity [23]. Taken together, BMI appears in-adequate as an indicator of adiposity in individuals who are disabled, as disability can lead to changes in fat distribution and body composition. Reduced muscle activity results in muscle volume loss and atrophy that lead to lower BMI [24], while the accumulation of fat in the abdomen increases waist circumference [25]. Furthermore, individuals with a normal BMI and central obesity were shown to have a higher death rate compared to overweight/obese individuals [25,26,27], suggesting that central obesity in itself predisposes to a higher mortality risk.

We considered several variables as possible confounders of associations of disability severity with body measurements. Our findings showed no correlations of EDSS groups with disease duration. Similarly, the number of steroid treatments corrected to disease duration was not associated with neurological disability, and we did not find a steroid-accumulating effect on EDSS over time. Glucocorticosteroids have been re-ported to promote whole-body insulin resistance via visceral adipogenesis, mobilization and release of free fatty acids into the circulation, and the development of hepatic steatosis [28]. Conceivably, the short-term customary treatment of administrating high-dose methylprednisolone intravenously for 5 consecutive days during acute MS relapses did not result in long-term weight gain and did not lead to obesity. Our observation is in agreement with reports that serious adverse events were not observed after high-dose glucocorticoid treatment. [29].

Engagement in physical activity was also assessed as a possible confounder of as-sociations of disability severity with body measurements. Our findings showed no difference in physical engagement among participants with different EDSS scores. This finding supports previous data from our center that showed no relation between physical activity and obesity, as measured by BMI in 238 individuals with MS with a mean ± SD EDSS score of 2.5 ± 1.7 [30].

This study has some limitations. We do not have longitudinal data on the cumulative exposure to obesity, and we lack information regarding dietary habits. Additionally, anthropometric measures used in the study did not include an assessment of fat percentage. Furthermore, engagement in physical activity was self-reported, and in-tensity and type of activity were not assessed. A strength of this study is the accurate assessments of neurological disability using the EDSS score by a trained neurologist rather than the use of self-reported assessment. Moreover, weight, height and waist circumference were measured systematically rather than based on self-reported measures; the latter tending to under-reported weight and BMI, and over-reported height [31].

In conclusion, our study found that among individuals with MS, disability is associated with central obesity but not with BMI. Adults with MS and higher neurological disability tend to have higher waist circumference and are therefore subjected to in-creased risk of cardiovascular morbidity. Based on our findings, we recommend the measurement of waist circumference for individuals with MS as a vital sign in clinical practice.

## Figures and Tables

**Table 1:** Characteristics of individuals with multiple sclerosis according to the different Expanded Disability Status Scale (EDSS) groups.

	EDSS ≥3 *n* = 130	EDSS 3.0–4.0 *n* = 31	EDSS 4.5–5.5 *n* = 24	EDSS 6.0 *n* = 44	EDSS ≥6.5 *n* = 31	*p* Value
Age (years)	55.8 ± 0.5	54.7 ± 0.9	54.1 ± 1.2	56.6 ± 0.9	56.9 ± 1.2	0.139
Disease duration (years)	18.2 ± 0.9	17.1 ± 1.6	15.5 ± 1.7	19.4 ± 1.5	19.8 ± 2.1	0.488
Steroid courses/disease duration	0.42 ± 0.05	0.22 ± 0.06	0.45 ± 0.13	0.48 ± 0.10	0.52 ± 0.12	0.18
BMI (kg/m^2^)	26.6 ± 0.5	25.7 ± 0.9	27.2 ± 1.4	25.8 ± 0.8	28.2 ± 1.3	0.596
Overweight (%)	35	42	29	30	39	0.207
Obese (%)	18	10	21	18	26	0.591
Weight (kg)	72.9 ± 1.4	70.3 ± 2.0	73.9 ± 3.7	70.7 ± 2.3	77.9 ± 3.5	0.408
Waist circumference (cm)	97.9 ± 1.3	94.6 ± 1.9	96.6 ± 3.2	96.6 ± 2.2	104.4 ± 3.0	0.045
Abnormal WC in males (%)	35	11	33	45	45	0.113
Abnormal WC in females (%)	66	59	61	61	85	0.113
Physical activity h/week n (%)						
None	56 (43.1)	11 (35.5)	8 (33.3)	20 (45.5)	17 (54.8)	0.596
<2.5	33 (25.4)	7 (22.6)	8 (33.3)	13 (29.5)	5 (16.1)	
2.5–5	25 (19.2)	11 (35.5)	5 (20.8)	5 (11.4)	4 (12.9)	
≥5	16 (12.3)	2 (6.4)	3 (12.5)	6 (13.6)	5 (16.1)	

The data are presented as mean ± standard deviation unless stated otherwise. BMI, basal metabolic rate; EDSS, expanded disability status scale; WC, waist circumference.

## Data Availability

The data presented in this study are available on request from the corresponding author.
